# Epinephrine delivery via EpiPen^®^ Auto-Injector or manual syringe across participants with a wide range of skin-to-muscle distances

**DOI:** 10.1186/s13601-020-00326-x

**Published:** 2020-06-10

**Authors:** Margitta Worm, DucTung Nguyen, Russ Rackley, Antonella Muraro, George Du Toit, Tracey Lawrence, Hong Li, Kurt Brumbaugh, Magnus Wickman

**Affiliations:** 1 Division of Allergy and Immunology, Department of Dermatology and Allergy, Charité Universitätsmedizin, Berlin, Germany; 2 Meda Pharma GmbH & Co KG, Bad Homburg vor der Hӧhe, Germany; 3 Mylan Inc, Canonsburg, PA USA; 4 Food Allergy Referral Centre, Department of Woman and Child Health, Padua University Hospital, Padua, Italy; 5 Children’s Allergy Service, Evelina London, Guy’s and St Thomas’ Hospital, London, UK; 6 Department of Women and Children’s Health, Pediatric Allergy, School of Life Course Sciences, Faculty of Life Sciences and Medicine, King’s College London, London, UK; 7 MRC & Asthma UK Centre in Allergic Mechanisms of Asthma, London, UK; 8 Centre for Clinical Research Sörmland, Uppsala University, Eskilstuna, Sweden

**Keywords:** Epinephrine, Adrenaline, Auto-injectors, Obesity, Body mass index, Intramuscular injections, Pharmacokinetics, Anaphylaxis, Skin-to-muscle distance, Needle length

## Abstract

**Background:**

Intramuscular (IM) injection of epinephrine (adrenaline) at the mid-anterolateral (AL) thigh is the international standard therapy for acute anaphylaxis. Concerns exist regarding implications of epinephrine auto-injector needles not penetrating the muscle in patients with greater skin-to-muscle-distances (STMD).

**Methods:**

This open-label, randomized, crossover study investigated pharmacokinetics and pharmacodynamics following injection of epinephrine in healthy volunteers. Individuals were stratified by maximally compressed STMD (low, < 15 mm; moderate, 15–20 mm; high, > 20 mm). Participants received epinephrine injections via EpiPen^®^ Auto-Injector (EpiPen; 0.3 mg/0.3 mL) or IM syringe (0.3 mg/0.3 mL) at mid-AL thigh or received saline by IM syringe in a randomized order. Eligible participants received a fourth treatment (EpiPen [0.3 mg/0.3 mL] at distal-AL thigh). Model-independent pharmacokinetic parameters and pharmacodynamics were assessed.

**Results:**

There were numerical trends toward higher peak epinephrine concentrations (0.52 vs 0.35 ng/mL; geometric mean ratio, 1.40; 90% CI 117.6–164.6%) and more rapid exposure (time to peak concentration, 20 vs 50 min) for EpiPen vs IM syringe at mid-AL thigh across STMD groups. Absorption was faster over the first 30 min for EpiPen vs IM syringe (partial area under curve [AUC] over first 30 min: geometric mean ratio, 2.13; 90% CI 159.0–285.0%). Overall exposure based on AUC to the last measurable concentration was similar for EpiPen vs IM syringe (geometric mean ratio, 1.13; 90% CI 98.8–129.8%). Epinephrine pharmacokinetics after EpiPen injection were similar across STMD groups. Treatments were well tolerated.

**Conclusions:**

Epinephrine delivery via EpiPen resulted in greater early systemic exposure to epinephrine vs IM syringe as assessed by epinephrine plasma levels. Delivery via EpiPen was consistent across participants with a wide range of STMD, even when the needle may not have penetrated the muscle.

*Trial registrations*This trial was registered with the German Clinical Trials Register (DRKS-ID: DRKS00011263; secondary ID, EudraCT 2016-000104-29) on 23 March 2017.

## Background

The international standard therapy for acute anaphylaxis is the prompt intramuscular (IM) injection of epinephrine in the mid-anterolateral (AL) thigh [1–3]. This can be achieved with the use of epinephrine auto-injectors such as EpiPen^®^ (epinephrine injection) Auto-Injector (EpiPen; Mylan Specialty L.P., Canonsburg, PA), which delivers a bolus of epinephrine through the use of a spring-loaded cartridge [2, 4]. As anaphylactic reactions can occur within minutes after exposure to an allergen, rapid and accurate epinephrine delivery via IM injections is crucial for reducing fatal reactions [5]. Although the needle length of EpiPen (approximately 16 mm) may reach and penetrate the thigh muscle in most people, rising obesity levels have raised concerns regarding a possible lack of efficacy if auto-injector needles fail to penetrate beyond the subcutaneous (SC) layer of fat and into muscle tissue in individuals with a greater skin-to-muscle distance (STMD) [2, 6, 7].

Other factors that may affect epinephrine delivery include the force and speed of delivery of epinephrine through the needle. This force and speed depends on the needle gauge and length, as well as the characteristics of the injection device (e.g., auto-injector mechanisms vs manual syringe injection) [4]. A previous study in a pig model demonstrated that epinephrine delivery via an auto-injector results in greater dispersion and faster uptake of epinephrine than manual delivery via syringe [4]; however, its implications regarding epinephrine administration via EpiPen in humans are unclear.

The present study was performed to assess whether EpiPen can provide systemic delivery of epinephrine in healthy volunteers with a wide range of STMD compared with manual syringe injections of epinephrine with customized needle lengths to ensure IM injection.

## Methods

### Study design and inclusion criteria

This exploratory study was an open-label, randomized, crossover study assessing pharmacokinetics (PK), pharmacodynamics (PD), and safety after epinephrine administration via EpiPen or IM syringe in people with a wide range of STMD. This study was designed using advice from the European Medicines Agency (EMA) Scientific Advice Working Party. This study was agreed upon as being exploratory in nature but was designed to utilize methodology recommended for bioequivalence studies. This study received Institutional Review Board approval, was conducted in accordance with the Declaration of Helsinki, and was registered as EudraCT 2016-000104-29.

Participants were healthy volunteers aged 18 to 55 years with a body mass index (BMI) ranging from 18 to 40 kg/m^2^. Anthropometric measurements, including STMD at the mid- and distal-AL thigh, were collected at screening. Skin-to-muscle distance was measured as the depth from the surface of the skin to the surface of the vastus lateralis muscle, which was measured with ultrasound imaging on the participant’s dominant side, under both minimum and maximum compression. Minimum compression was defined as the pressure induced by the weight of the ultrasound sensor with no additional pressure enforced other than what was required to ensure an adequate reading. Maximum compression was defined as the operator pressing the ultrasound sensor with maximum force against the femur bone and withdrawing only as needed to obtain a clear ultrasound image. Skin-to-muscle distances were measured in triplicate for both minimum and maximum levels of compression. The average STMD values at the mid-AL thigh under maximum compression were used for stratification into 3 sex-balanced groups, defined as low (< 15 mm), moderate (15–20 mm), or high (> 20 mm) STMD. The study was designed to enroll 12 participants for each STMD group, balanced by sex, with 12 evaluable participants being considered the minimum number for bioequivalence studies [8]. Written informed consent was obtained from all participants before screening.

### Treatment administration

Each participant received 3 different unblinded injections (epinephrine via EpiPen [0.3 mg/0.3 mL], IM epinephrine via syringe [0.3 mg/0.3 mL], or saline via syringe [0.3 mL]) at the mid-AL thigh in a randomized order, with 24-h washout periods between each injection. Participants with a skin-to-bone distance (STBD) ≥ 20 mm at the distal-AL thigh received a fourth injection (epinephrine via EpiPen [0.3 mg/0.3 mL]) at that site. Participants with an STBD < 20 mm and all participants in the low-STMD group were excluded from injection at the distal-AL thigh for safety reasons. To ensure IM injection, the needle lengths for the manual IM syringe were individualized for each participant to be approximately 30% longer than the participant’s mean STMD at minimum compression. Efforts were made to minimize injection variability between groups by standardizing preparation of the participants for injection (e.g., positioning, marking of injection site area), having a minimal number of physicians administering injections, and following standard IM injection procedures. The mean (range) needle lengths for the low-, moderate-, and high-STMD groups were 19.4 (12–30), 27.9 (25–40), and 39.1 (30–40) mm, respectively. Per the protocol, 22-gauge needles were used if available. However, this was not always logistically possible because of a finite number of commercially available needles; if 22 gauge was not available, the nearest gauge available for the required needle length was used (median needle gauge, 23; range, 22–27; Additional file 1).

### Study measurements

The primary objective of this study was to estimate differences in epinephrine PK after administration of epinephrine 0.3 mg via EpiPen or IM syringe at the mid-AL thigh. As epinephrine is endogenous, saline administration via IM syringe was included as a control. For each study period, 6-mL blood samples were collected into EDTA vacutainer tubes at predefined time points before and after epinephrine administration. Blood samples were then centrifuged under refrigeration to separate plasma, and 2 mL of the plasma sample was transferred into a polypropylene tube containing 50 μL of sodium metabisulfite. The plasma samples were frozen immediately at − 70 °C and kept in a freezer until analysis. A 96-well solid-phase extraction and high-performance liquid chromatography with tandem mass spectrometric detection was used to assess epinephrine concentrations with a limit of quantification of 0.05 ng/mL and a linear range from 0.05 to 10.0 ng/mL. Exposure to epinephrine was inferred on the basis of model-independent PK parameters, including peak epinephrine plasma concentrations (C_peak_), time to C_peak_ (t_peak_), area under the epinephrine plasma concentration–time curve (AUC) to the last measurable concentration (AUC_0-t_), and partial AUC at 6, 15, and 30 min. These parameters were calculated using noncompartmental techniques and compared across injection types (EpiPen vs IM syringe) and STMD groups. Pharmacodynamic parameters, including systolic blood pressure, diastolic blood pressure, and heart rate, were measured at predefined times before and after dosing. Participants were asked open-ended queries regarding the presence or absence of adverse events (AEs) every 8 to 12 h during their stay in the clinic.

Correlations of PK and PD parameters with anthropometric measurements were assessed by Pearson r and Kendall tau. Anthropometric measurements were assessed during the screening period and included BMI; height; weight; STMD at mid- and distal-AL thigh; circumference of the thigh, hip, waist, and neck; and skin fold at the mid thigh, distal thigh, abdomen, and chest.

### Statistical analyses

The statistical analyses for this exploratory study design were adapted on the basis of guidelines for bioequivalence studies (e.g., the use of 90% confidence intervals [CIs] rather than *P* values). Statistical analyses were performed on PK and PD parameters using crossover analysis of covariance with sequence, subject (sequence), period, treatment, STMD group, and sex as fixed effects and baseline values as continuous covariates for the first 3 periods. The 90% CIs were calculated for PK-pairwise treatment comparisons, and 95% CIs were calculated for both PK- and PD-pairwise treatment comparisons. Geometric mean ratios were used to compare PK parameters, and PD parameters were analyzed in a similar way for consistency. Although this study was exploratory and not powered a priori for assessing statistical superiority, interventions were considered to be bioequivalent to a comparator if the 90% CI for the relative mean fell within 80% to 125% for C_peak_ and AUC measurements [8]. Likewise, if the 90% CI for the relative mean fell completely outside the 80% to 125% range, interventions were not considered to be bioequivalent. For correlation between anthropometric measurements and epinephrine PK parameters, Pearson r and Kendall tau correlation coefficient were assessed and tested for significance. For measuring changes in PD parameters (i.e., heart rate and systolic and diastolic blood pressure), changes relative to median baseline values were assessed using analysis of covariance with a significance level of *P *< 0.05.

## Results

### Baseline demographics

Healthy participants were included in the study, with a target enrollment of 36 participants. Only 5 male participants met the inclusion criteria for the high-STMD group within the study timeline; therefore, 35 participants were included. Participants were stratified into 3 groups on the basis of STMD under maximum compression and sex (low STMD, < 15 mm, n = 12, 6 females and 6 males; moderate STMD, 15–20 mm, n = 12, 6 females and 6 males; and high STMD, > 20 mm, n = 11, 6 females and 5 males). Baseline demographics (age, weight, height, STMD) are presented in Table 1. Average BMI values and weight tended to be higher in groups with greater STMD. Other baseline variables (i.e., age, gender, height) were well balanced between groups, though females in each STMD group tended to be older than their male counterparts, particularly in the low-STMD group. Most participants (22/35; 63%) in this study were obese, with an overall mean (standard deviation) BMI of 30.4 (5.97) kg/m^2^, which is higher than in the general population [9].

**Table 1:** Baseline Demographics of Healthy Volunteers

Parameter, mean (SD)^a^	Low STMD (< 15 mm; n = 12)^b^			Moderate STMD (15–20 mm; n = 12)^b^			High STMD (> 20 mm; n = 11)^b^		
	Males (n = 6)	Females (n = 6)	All (n = 12)	Males (n = 6)	Females (n = 6)	All (n = 12)	Males (n = 5)	Females (n = 6)	All (n = 11)
Age, year	36.2 (4.2)	46.0 (5.5)	41.1 (7.0)	34.5 (6.0)	38.8 (10.6)	36.7 (8.5)	32.8 (11.4)	39.0 (11.4)	36.2 (11.3)
Weight, kg	103.5 (25.7)	63.6 (7.6)	83.5 (27.6)	107.7 (10.7)	82.3 (10.4)	95.0 (16.6)	122.4 (6.4)	85.0 (15.3)	102.0 (22.7)
Height, cm	177.5 (8.5)	172.8 (5.3)	175.2 (7.2)	177.5 (8.9)	171.7 (9.8)	174.6 (9.4)	184.2 (6.5)	164.0 (6.1)	173.2 (12.1)
BMI, kg/m^2^	32.4 (5.7)	21.3 (2.4)	26.8 (7.1)	32.2 (2.1)	28.1 (4.5)	31.1 (4.6)	36.1 (2.2)	31.4 (3.8)	33.6 (3.9)
Mid STMD, min compression, mm	11.6 (4.1)	13.0 (6.4)	12.3 (5.2)	18.1 (2.1)	18.4 (1.8)	18.2 (1.9)	24.8 (1.9)	26.1 (3.2)	25.5 (2.6)
Mid STMD, max compression, mm	10.1 (3.8)	10.1 (4.0)	10.1 (3.7)	16.5 (1.1)	16.2 (1.5)	16.4 (1.2)	20.8 (0.3)	23.2 (3.0)	22.1 (2.5)
Distal STMD, min compression, mm	10.6 (4.2)	10.9 (4.2)	10.7 (4.0)	15.3 (2.7)	16.1 (3.3)	15.7 (2.9)	20.3 (2.7)	20.0 (3.8)	20.2 (3.2)
Distal STMD, max compression, mm	9.4 (4.0)	9.6 (3.2)	9.5 (3.5)	13.4 (1.9)	13.7 (2.6)	13.5 (2.2)	16.4 (2.7)	17.0 (3.2)	16.7 (2.8)
IM syringe needle length, (range), mm^c^	18.2 (12–25)	20.7 (12–30)	19.4 (12–30)	29.2 (25–40)	26.7 (25–30)	27.9 (25–40)	40.0 (40–40)	38.3 (30–40)	39.1 (30–40)

*BMI* body mass index, *max* maximum, *min* minimum, *SD* standard deviation, *STMD* skin-to-muscle distance

^a^Unless otherwise noted

^b^Based on maximum compression of STMD

^c^Values are the mean; needle length for EpiPen Auto-Injector was approximately 16 mm for all STMD groups

### Pharmacokinetics

Baseline epinephrine levels were below the quantitation range of the assay (< 0.05 ng/mL), so no baseline correction was performed for calculation of PK parameters. Similarly, injection of 0.9% isotonic saline resulted in negligible measurable epinephrine response and therefore was not included in any statistical comparisons of PK parameters.

**Fig. 1:**
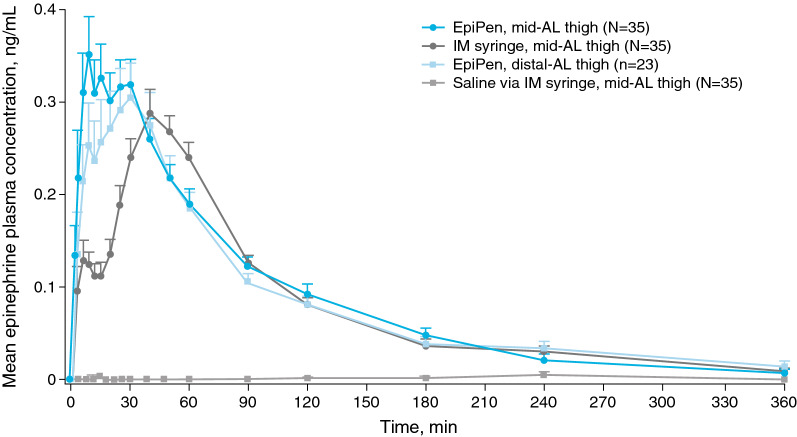
Epinephrine plasma concentrations after administration via EpiPen or syringe at the mid-AL or distal-AL thigh. Mean epinephrine plasma concentrations after epinephrine administration via EpiPen at the mid-AL thigh (N = 35), epinephrine administration via IM syringe at the mid-AL thigh (N = 35), saline administration via IM syringe at the mid-AL thigh (N = 35), and epinephrine administration via EpiPen at the distal-AL thigh (n = 23). Error bars are the standard error of the mean. *AL* anterolateral, *EpiPen* EpiPen Auto-Injector, *IM* intramuscular

Epinephrine administrations via EpiPen vs IM syringe were compared at the mid-AL thigh. EpiPen resulted in numerically higher C_peak_ values vs IM syringe (0.52 vs 0.35 ng/mL, respectively; geometric mean ratio, 1.40; 90% CI 117.6–164.6%; Fig. 1; Additional file 2). Epinephrine also reached maximum concentrations more rapidly via EpiPen vs IM syringe, as evidenced by a shorter median t_peak_ (20 vs 50 min, respectively). However, overall exposure (AUC_0-t_) to epinephrine was similar (though marginally higher) for EpiPen vs IM syringe at the mid-AL thigh (30.0 vs 26.1 ng min/mL, respectively; geometric mean ratio, 1.13; 90% CI 98.8–129.8%).

A post hoc analysis of partial AUCs indicated greater early exposure to epinephrine via EpiPen vs IM syringe at the mid-AL thigh over the first 30 min after injection (geometric mean ratio, 2.13; 90% CI 159.0–285.0%). Similar observations were made for partial AUC at 6 min postinjection (geometric mean ratio, 2.22; 90% CI 136.9–361.3%) and partial AUC at 15 min postinjection (geometric mean ratio, 2.16; 90% CI 142.2–327.8%). These comparisons emphasize a faster absorption of epinephrine via EpiPen compared with IM syringe.

It was also investigated whether administration via EpiPen at an alternate injection site (the distal-AL thigh) for the moderate- and high-STMD groups would lead to an increase in systemic exposure to epinephrine because of an increased likelihood for IM injection. However, administration via EpiPen at the distal-AL thigh in these groups resulted in a marginally lower C_peak_ than administration at the mid-AL thigh (0.41 vs 0.52 ng/mL; geometric mean ratio, 0.77; 90% CI 63.1–93.8%; Fig. 1). Median t_peak_ was also slightly slower after administration at the distal- vs mid-AL thigh (25 vs 20 min), and total exposure was similar (though marginally lower) for the distal- vs mid-AL thigh (28.1 vs 30.0 ng min/mL, respectively; geometric mean ratio, 0.91; 90% CI 77.8–107.4%).

Additional comparisons of the moderate- and high-STMD groups showed that epinephrine administration via EpiPen at the distal-AL thigh also resulted in more rapid epinephrine exposure than IM syringe at the mid-AL thigh, as evidenced by a shorter median t_peak_ (25 vs 50 min). The average C_peak_ after administration via EpiPen at the distal-AL thigh was similar (though marginally higher) than observed with administration via IM syringe at the mid-AL thigh (0.41 vs 0.35 ng/mL, respectively; geometric mean ratio, 1.07; 90% CI 87.8–130.6%). Overall epinephrine exposure was similar between these 2 administration methods (28.1 vs 26.1 ng min/mL, respectively; geometric mean ratio, 1.04; 88.1–121.6%).

### Injection types across STMD groups

**Fig. 2:**
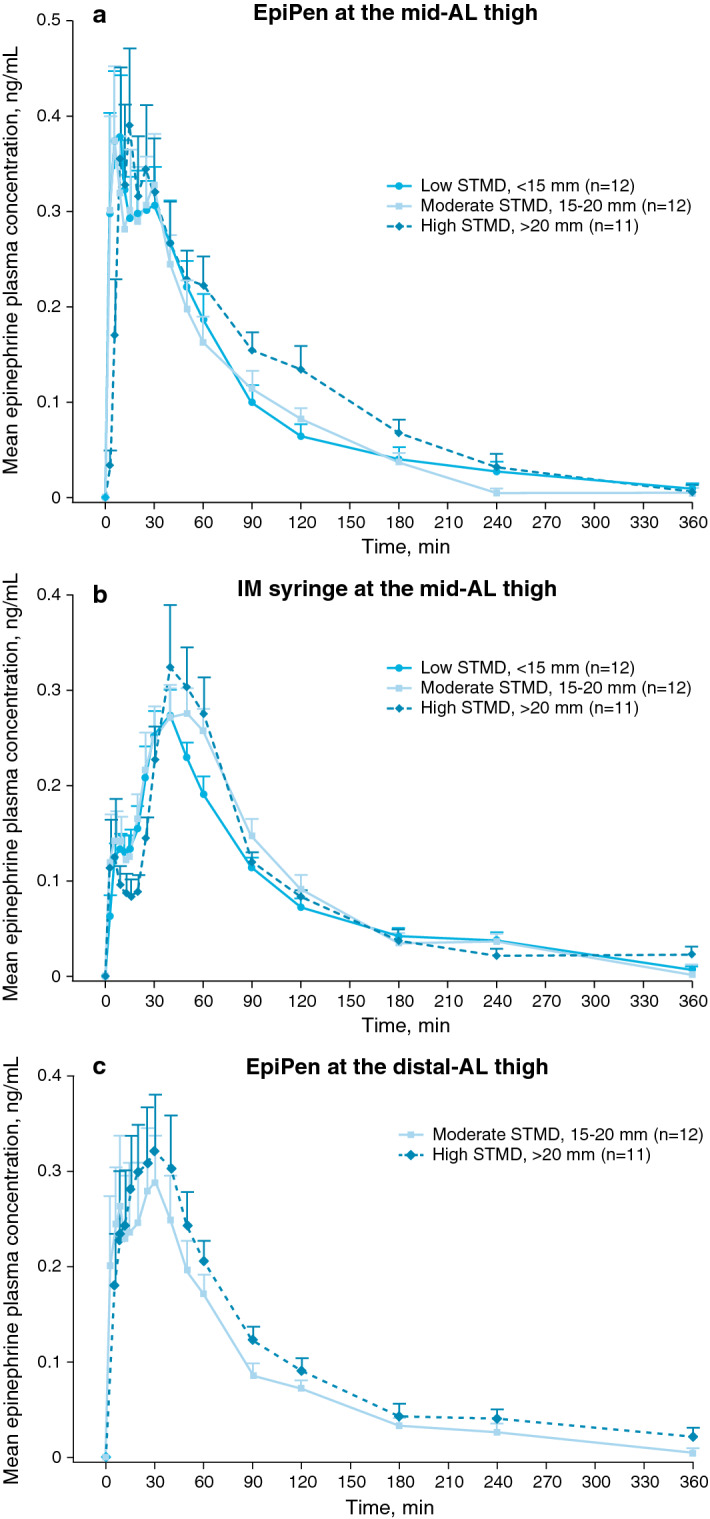
Epinephrine plasma concentrations stratified by STMD group for different injection types and locations. Mean epinephrine plasma concentrations in participants with low (< 15 mm), moderate (15–20 mm), and high (> 20 mm) STMD after epinephrine administration with **(a)** EpiPen at the mid-AL thigh, **(b)** IM syringe at the mid-AL thigh, or **(c)** EpiPen at the distal-AL thigh. The low-STMD group did not receive epinephrine administration via EpiPen at the distal-AL thigh because of safety considerations. Error bars are the standard error of the mean. *AL* anterolateral, *EpiPen* EpiPen Auto-Injector, *IM* intramuscular, *STMD* skin-to-muscle distance

Because the length of the EpiPen needle (approximately 16 mm) may be insufficient to reach the muscle layer at the mid-AL thigh in patients with greater-than-average STMD, it was investigated whether epinephrine exposure differs between individuals with a wide range of STMD. Although each group had a different range of STMD, epinephrine exposure after administration via EpiPen at the mid-AL thigh was similar across all 3 STMD groups (Fig. 2a; Table 2). Females tended to have greater C_peak_ values than males within STMD groups after administration via EpiPen. Post hoc analyses revealed no significant differences between STMD groups in partial AUC values at 6, 15, or 30 min after administration via EpiPen at the mid-AL thigh, reflecting a similar time course across a wide range of STMD. Mean plasma concentrations over time were also similar across STMD groups for IM syringe (Fig. 2b) and for moderate- and high-STMD groups receiving EpiPen at the distal-AL thigh (Fig. 2c).

**Table 2:** Epinephrine Pharmacokinetic Parameters for Each Treatment Group Stratified by STMD and Sex

Parameter, mean (SD)^a^	Low STMD (< 15 mm, n = 12)^b^			Moderate STMD (15–20 mm, n = 12)^b^			High STMD (> 20 mm; n = 11)^b^		
	Male (n = 6)	Female (n = 6)	All (n = 12)	Male (n = 6)	Female (n = 6)	All (n = 12)	Male (n = 5)	Female (n = 6)	All (n = 11)
EpiPen at the mid-AL thigh (n = 35)									
C_peak_, ng/mL	0.40 (0.10)	0.64 (0.37)	0.52 (0.29)	0.48 (0.30)	0.52 (0.23)	0.50 (0.25)	0.42 (0.21)	0.63 (0.31)	0.53 (0.28)
AUC_0-t_, ng min/mL	27.3 (10.4)	28.8 (8.9)	28.1 (9.3)	20.9 (7.4)	31.7 (15.9)	26.3 (13.1)	31.1 (13.3)	40.3 (13.1)	36.1 (13.4)
Median t_peak_ (range), min	17 (9–30)	5 (3–60)	9 (3–60)	4.5 (2–30)	15 (3–39)	10.5 (2–39)	30 (24–120)	24.5 (9–60)	30 (9–120)
IM syringe at the mid-AL thigh (n = 35)									
C_peak_, ng/mL	0.25 (0.09)	0.36 (0.10)	0.31 (0.10)	0.32 (0.15)	0.37 (0.07)	0.35 (0.11)	0.27 (0.10)	0.51 (0.24)	0.40 (0.22)
AUC_0-t_, ng min/mL	20.1 (8.7)	28.8 (6.8)	24.4 (8.7)	25.7 (8.7)	28.1 (10.1)	26.9 (9.1)	22.6 (13.1)	30.6 (13.8)	27.0 (13.4)
Median t_peak_ (range), min	50 (25–60)	40 (25–50)	40 (25–60)	49.5 (3–60)	45 (6–50)	45 (3–60)	50 (30–60)	50 (6–60)	50 (6–60)
EpiPen at the distal-AL thigh (n = 23)									
C_peak_, ng/mL	–	–	–	0.36 (0.26)	0.49 (0.32)	0.42 (0.28)	0.30 (0.11)	0.46 (0.21)	0.39 (0.19)
AUC_0-t_, ng min/mL	–	–	–	22.5 (6.9)	26.9 (15.5)	24.7 (11.7)	25.1 (10.4)	37.4 (11.9)	31.8 (12.4)
Median t_peak_ (range), min	–	–	–	29.5 (3–60)	25 (9–40)	25 (3–60)	30 (25–60)	9 (6–40)	25 (6–60)

*AL* anterolateral, *AUC*_*0-t*_ area under the epinephrine plasma concentration–time curve to the last measurable concentration, *C*_*peak*_ peak epinephrine plasma concentration, *EpiPen* EpiPen Auto-Injector, *IM* intramuscular, *SD* standard deviation, *STMD* skin-to-muscle distance, *t*_*peak*_ time to C_peak_

^a^Unless otherwise noted

^b^Based on maximum compression of STMD

**Fig. 3:**
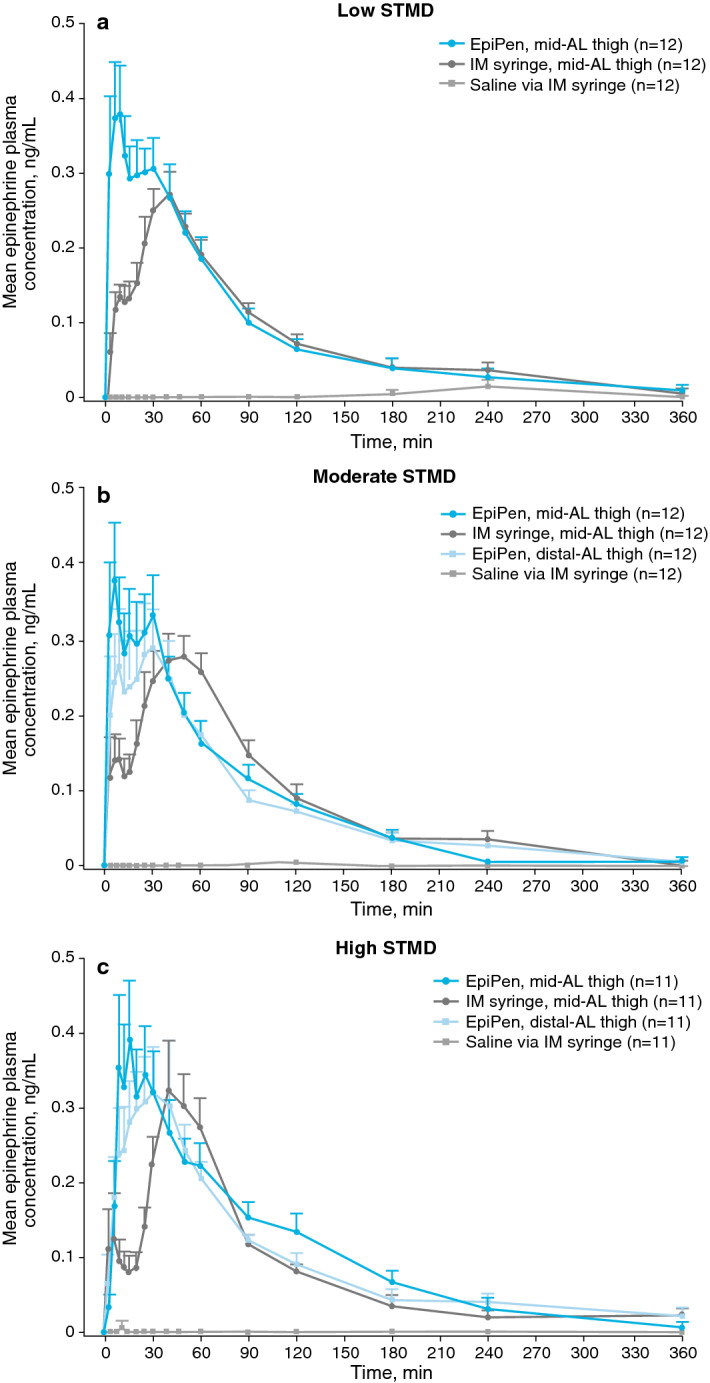
Epinephrine plasma concentrations stratified by injection type and location for low, moderate, and high STMD. Mean epinephrine plasma concentrations in participants with **(a)** low (< 15 mm), **(b)** moderate (15–20 mm), and **(c)** high (> 20 mm) STMD after different administration methods of epinephrine. The low-STMD group did not receive epinephrine administration via EpiPen at the distal-AL thigh because of safety considerations. Error bars are the standard error of the mean. *AL* anterolateral, *EpiPen* EpiPen Auto-Injector, *IM* intramuscular, *STMD* skin-to-muscle distance

Similar to trends previously observed, partial AUC analysis indicated more rapid delivery of epinephrine at the mid-AL thigh within the first 30 min via EpiPen vs IM syringe in the low-STMD group (geometric mean ratio, 2.09; 90% CI 148.0–295.9%; Fig. 3a), the moderate-STMD group (geometric mean ratio, 1.64; 90% CI 98.7–273.4%; Fig. 3b), and the high-STMD group (geometric mean ratio, 2.90; 90% CI 155.1–543.2%; Fig. 3c). Partial AUC at 6 and 15 min for epinephrine administration via EpiPen was comparable with or greater than that of IM syringe and afterwards trended toward a more rapid epinephrine absorption via EpiPen vs IM syringe for all STMD groups.

### Pharmacodynamics

Heart rate and blood pressure measurements were similar between STMD groups for each treatment, although these data were highly variable. Administration of epinephrine by either EpiPen or IM syringe led to significantly elevated heart rates within 5 min of injection in the overall population. These results were generally consistent across STMD groups. Epinephrine administration via EpiPen vs IM syringe at the mid-AL thigh resulted in higher maximum heart rates (76.9 vs 72.6 beats/min, respectively; geometric mean ratio, 1.05; 95% CI 102.2–108.4%) and shorter time to maximum heart rates (33.8 vs 60.4 min, respectively; geometric mean ratio, 0.66; 95% CI 20.8–88.6%). Similar trends were observed when epinephrine was administered via EpiPen at the distal-AL thigh vs IM syringe at the mid-AL thigh for both maximum heart rates (78.7 vs 72.6 beats/min, respectively; geometric mean ratio: 1.03; 95% CI 99.4–106.6%) and time to maximum heart rates (31.2 vs 60.4 min, respectively; geometric mean ratio, 0.48; 95% CI 9.1–87.6%). Changes were similar for heart rates after epinephrine administration via EpiPen at the distal-AL thigh vs the mid-AL thigh (78.7 vs 76.9 beats/min, respectively; geometric mean ratio, 0.98; 95% CI 94.4–101.2%) and for time to maximum heart rates (31.2 vs 33.8 min, respectively; geometric mean ratio, 0.88; 95% CI 16.5–160.3%).

**Fig. 4:**
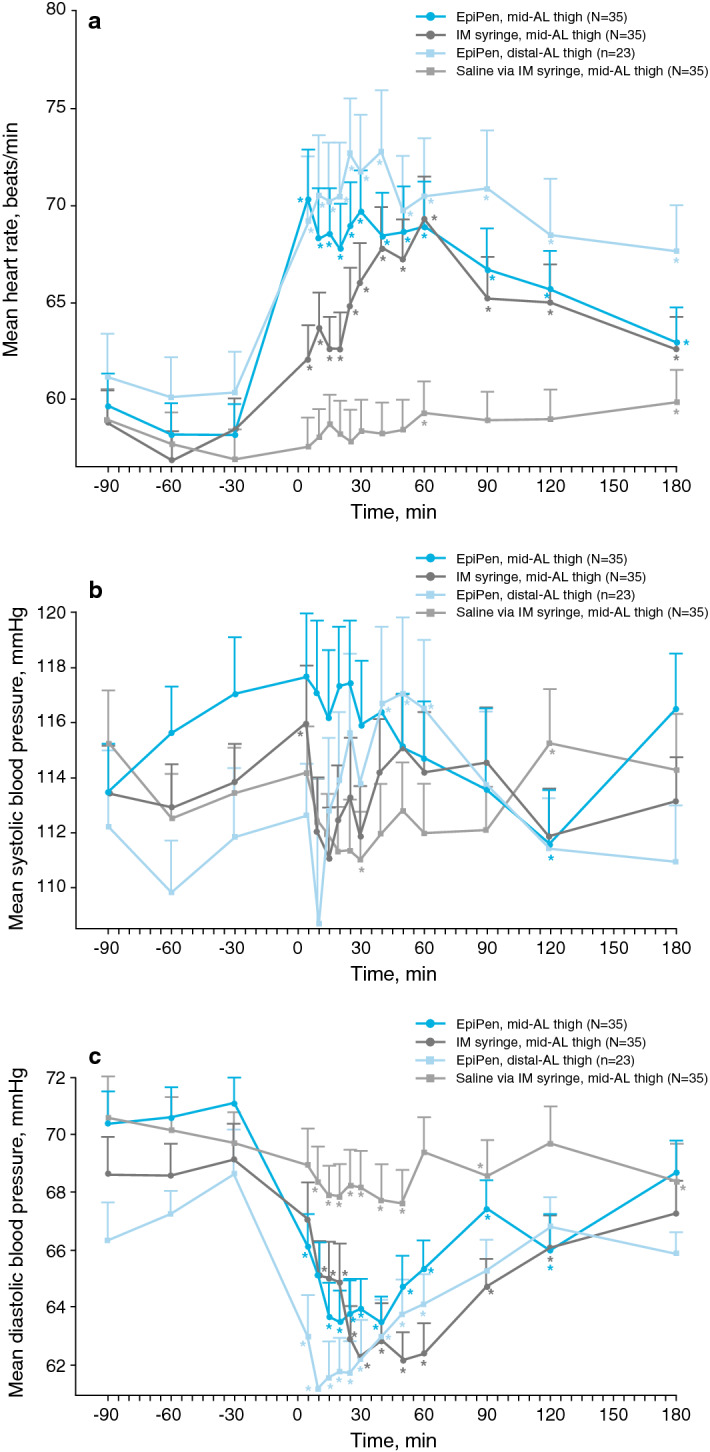
Pharmacodynamic measurements after administration of epinephrine or saline via different injection types and locations. Profiles of mean **(a)** heart rate, **(b)** systolic blood pressure, and **(c)** diastolic blood pressure at baseline and after epinephrine administration via EpiPen at the mid-AL thigh (N = 35), epinephrine administration via IM syringe at the mid-AL thigh (N = 35), saline administration via IM syringe at the mid-AL thigh (N = 35), and epinephrine administration via EpiPen at the distal-AL thigh (n = 23). Asterisks reflect significant changes (*P *< 0.05) relative to median baseline values. Error bars are the standard error of the mean. *AL* anterolateral, *EpiPen* EpiPen Auto-Injector, *IM* intramuscular

Although there were no clinically meaningful correlations between epinephrine PK and PD parameters, the mean profile for heart rate across treatments (Fig. 4a) followed similar trends to those observed with plasma levels of epinephrine (Fig. 1), in which heart rate changes were greater in magnitude and peak heart rates occurred more rapidly via EpiPen at the mid- and distal-AL thigh vs IM syringe. Heart rates were significantly elevated compared with median baseline heart rates for all 3 active treatments. In general, systolic blood pressure profiles demonstrated limited changes from baseline, and most of these changes were insignificant and did not appear to be similar to changes observed in epinephrine plasma levels (Fig. 4b). The profiles in diastolic blood pressure after injections were inversely similar to trends in epinephrine levels, though these trends were less pronounced than with heart rate measures (Fig. 4c). Significant changes in diastolic blood pressure were observed 5 min after EpiPen administration at the mid- and distal-AL thigh and 10 min after IM syringe injection. These trends in diastolic blood pressure changes were similar across STMD groups.

### Anthropometric measurements and PK parameters

To further investigate whether epinephrine exposure was influenced by participant characteristics, the correlations between anthropometric measurements and PK parameters were assessed using Pearson r and Kendall tau for EpiPen at the mid-AL thigh. Anthropometric measurements assessed included BMI; height; weight; STMD at mid- and distal-AL thigh; circumference of the thigh, hip, waist, and neck; and skin fold at the mid thigh, distal thigh, abdomen, and chest. In general, there were no clinically meaningful correlations between anthropometric measurements and epinephrine PK parameters (i.e., absolute value of correlation coefficients r and tau were not > 0.5), although both coefficients had significant *P* values (*P *< 0.05) for some comparisons. However, these results may have limited interpretability due to the small sample size for these correlations (n ≤ 35 for each correlation).

### Safety

The administered treatments were well tolerated and no participants were withdrawn from the study. Twenty-four participants experienced a total of 73 AEs (Table 3). All AEs were mild to moderate in severity and no serious AEs were reported. Palpitations were the most frequently reported AEs for EpiPen at the mid-AL thigh (17.1% [6/35]) and distal-AL thigh (34.8% [8/23]). The most frequently reported AEs for IM syringe were headache (11.4% [4/35]) and palpitations (11.4% [4/35]). The most frequently reported AE for saline injection was headache (8.6% [3/35]).

**Table 3:** Adverse Events

	Mid-AL thigh			Distal-AL thigh
	Epinephrine via EpiPen (N = 35)	Epinephrine via IM syringe (N = 35)	Saline via IM syringe (N = 35)	Epinephrine via EpiPen (n = 23)
Total AEs, n	27	25	7	15
AEs considered related to treatment (> 3% in any treatment group), n (%)^a^				
Palpitations	6 (17.1)	4 (11.4)	0	7 (30.4)
Feeling abnormal	4 (11.4)	2 (5.7)	0	2 (8.7)
Feeling hot	3 (8.6)	1 (2.9)	0	0
Tremor	3 (8.6)	1 (2.9)	0	1 (4.3)
Headache	1 (2.9)	2 (5.7)	1 (2.9)	0
Limb discomfort	1 (2.9)	1 (2.9)	0	1 (4.3)
Myalgia^b^	0	2 (5.7)	0	0

*AE* adverse event, *AL* anterolateral, *EpiPen* EpiPen Auto-Injector, *IM* intramuscular

^a^AEs are listed in order of incidence

^b^Myalgia was reported only in thigh muscle

## Discussion

Concerns have been raised that the length of epinephrine auto-injectors may be insufficient to penetrate the muscle layer of the mid-AL thigh in patients with high STMD, which may interfere with reliable IM injection of epinephrine for treatment of acute anaphylaxis. This study demonstrated that epinephrine administration with EpiPen resulted in higher peak plasma levels of epinephrine and more rapid systemic delivery during the first 30 min postinjection compared with manual IM syringe injections with individualized needle lengths selected to reach the muscle layer. Moreover, systemic epinephrine delivery was observed across individuals with varying STMD, including those with an STMD greater than the EpiPen needle length.

The ability of EpiPen to provide systemic delivery of epinephrine despite having a needle length insufficient to reach the muscle layer at the mid-AL thigh in some individuals is encouraging and supported by previous studies in preclinical models and healthy volunteers [4, 10]. However, the mechanisms underlying this ability are unclear. One possible explanation is that the force of delivery provided by the spring-loaded mechanism of EpiPen may enable the propulsion of epinephrine beyond the SC fat layer or promote greater contact between the injectate and the vascular bed, resulting in greater dispersion and systemic uptake of epinephrine [4, 10]. An alternative explanation may be that SC absorption of epinephrine is sufficient to drive systemic delivery. Indeed, SC epinephrine administration is recommended as a second-line treatment option for acute severe asthma if primary inhalational treatment is ineffective [11]. Although it has been previously suggested that SC administration of epinephrine is unlikely to penetrate the IM layer because of the impermeability of the deep fascia of the thigh [12], the possibility that SC absorption is sufficient for systemic delivery of epinephrine appears to be supported by a separate study that investigated epinephrine administration via an epinephrine auto-injector in overweight females (BMI, 26–34 kg/m^2^) who had similar peak exposure to epinephrine compared with males with normal BMI, despite adrenaline fluid depots being deposited subcutaneously in the vast majority of these females [10]. This previous study also noted a more delayed elevation in epinephrine plasma levels after Anapen^®^ (Lincoln Medical Ltd, Wiltshire, United Kingdom) injection in overweight females compared with males with normal BMI [10]; however, such a delay with EpiPen administration was not observed in our study in participants with higher STMD compared with participants with lower STMD. This could possibly be attributed to differences in device characteristics between Anapen and EpiPen (e.g., spring force, extended-needle length, needle gauge) [4]. Of note, the median t_peak_ in participants with high STMD receiving EpiPen at the mid-AL thigh was 30 min, which was numerically higher than the median t_peak_ observed for the low- and moderate-STMD groups (9 and 15 min, respectively). However, the median t_peak_ in the high-STMD group was even longer (50 min) after manual IM syringe injection with individualized needle lengths selected to reach the muscle layer. Similarly, partial AUC analyses revealed much greater systemic exposure to epinephrine in the high-STMD group during the first 30 min postinjection compared with manual IM syringe injections. These data suggest that EpiPen injection at the mid-AL thigh remains a preferred approach in patients with high STMD, even if the peak exposure to epinephrine is delayed in these patients compared with patients with lower STMD.

The differences between the auto-injector and IM syringe were more pronounced in the current study compared with a recent study by Duvauchelle et al. [10] However, it is notable that after IM syringe delivery, the PK profiles were similar between studies. Both studies reported an initial peak in epinephrine plasma concentrations after IM syringe injection at approximately 5 to 10 min after injection followed by a later, higher peak at approximately 50 min after injection. Moreover, reported C_peak_ values for IM syringe were comparable between studies (0.35 ng/mL in the current study; 0.40 ng/mL in Duvauchelle et al.). The main difference between these studies was a slightly greater contrast in C_peak_ values between the auto-injector and IM syringe, which could be due to differences in auto-injector device characteristics. The epinephrine PK profiles for auto-injector and IM syringe in both studies—particularly the relative magnitude of the initial peak after IM syringe injection—differ from epinephrine PK profiles from a previous report published in 2001 (Simons et al.) [13]. Notably, the initial peaks after injection via EpiPen or IM syringe were of comparable magnitude and timing in Simons et al., though C_peak_ values were approximately 20% lower for IM syringe vs EpiPen. Additionally, despite administering the same dose of epinephrine as the recent studies, the overall magnitudes of C_peak_ for both treatments were much higher in the Simons study (approximately 9.7–12.2 ng/mL) vs those of the current study and Duvauchelle et al. (approximately 0.3–0.5 ng/mL). The reasons for these differences between studies are unclear but could be due to differences in patient populations (e.g., sample size, sex, BMI), assay methodology, or other undetermined inter-study characteristics. Ultimately, it is the opinion of the authors that the similarity of PK results between the current study and the recent Duvauchelle et al. study support the conclusions of the current study that epinephrine injection via EpiPen results in higher peak epinephrine concentrations and more rapid systemic delivery compared with epinephrine delivery via manual IM syringe.

Reliable delivery of epinephrine via EpiPen across participants was also supported by changes in heart rate observed in this study, with EpiPen administration leading to significant elevations in heart rate within 5 min postinjection. Although there are limited studies assessing PD after epinephrine administration via EpiPen, the changes in heart rate we observed in healthy participants receiving 300-µg injections of epinephrine are believed to be directly related to systemic delivery of epinephrine [10, 14]. The absolute and relative changes in heart rate observed in this study after epinephrine administration via EpiPen and IM syringe were similar to those observed in a previous study assessing a different epinephrine auto-injector device [10]. Unexpectedly, statistically significant reductions from baseline in systolic and diastolic blood pressure were observed at some time points after epinephrine injection; however, the magnitude of these changes were low and unlikely to be clinically relevant, as significant reductions were also observed with saline. The mechanism underlying such reductions is unclear but could be attributed to a general vasovagal response.

A series of previous studies assessed whether various epinephrine auto-injectors had an elevated risk of SC injection in children or adults [15–18]. The authors concluded that a few epinephrine auto-injectors, including EpiPen, had an elevated risk of SC injection in some adults and children with higher STMD on the basis of semiquantitative grading. However, these conclusions were based on ultrasound or computed tomography measurements, needle lengths, and an estimated needle length threshold needed to reach the muscle layer, and no epinephrine injections or PK measurements were made in these studies. Although our current study did not directly assess whether epinephrine was deposited in the muscle layer with mid–AL EpiPen injection, systemic epinephrine exposure was observed across all STMD groups despite the study population being on average larger than the general population. Though our study did not directly address the conclusions of these studies that epinephrine may be deposited subcutaneously with EpiPen, the systemic epinephrine delivery observed in our study should help address the implications of these concerns previously raised that EpiPen needle length is insufficient to drive systemic exposure to epinephrine.

These results suggest that the design of EpiPen is sufficient for use across a broad clinical population. The needle lengths used for epinephrine administration via IM syringe in the current study for moderate- and high-STMD groups (Table 1) were equal to or greater than those recommended in emergency care settings by the UK Resuscitation Council anaphylaxis guide, which suggests 25-mm needles to 38-mm needles for some adults [19, 20]. According to recent updates from the World Health Organization [9], the mean age-standardized BMI of European adults is approximately 26.4 kg/m^2^. Participants included in this study had a mean (standard deviation) BMI of 30.4 (5.97) kg/m^2^, and these data support the ability of EpiPen to systemically deliver epinephrine to individuals with a wide range of STMD, including those with compressed STMD greater than the needle length. The ability of EpiPen to consistently deliver systemic epinephrine is further supported by the lack of strong correlations between PK parameters and anthropometric measurements, though interpretation of these correlations may be limited by the small sample size.

There are several limitations to this study. First, the sample size was somewhat small because of the exploratory nature of the study, which may limit the interpretability of statistical differences between groups, particularly given the variability in PK and PD measurements. However, the size was considered minimally adequate for assessing bioequivalence within groups. Second, this study was a single-center study, so the generalizability of these results to other geographic regions is unclear; however, we feel the distribution of BMI in this study was appropriate for the concerns this study was designed to address (i.e., previous concerns that EpiPen may be insufficient to provide systemic epinephrine delivery in individuals with high STMD). Third, although this study demonstrated rapid absorption kinetics of epinephrine after EpiPen administration vs IM syringe across different STMD groups, this study was conducted in healthy participants. Because there have been no randomized controlled studies of epinephrine PK in patients experiencing anaphylaxis, it is unclear how these epinephrine PK data would extrapolate to the efficacy of EpiPen in anaphylactic cases, including the relative efficacy compared with IM syringe [1]. Fourth, the IM syringe needles used for this study were selected on the basis of needle length, rather than needle gauge, to ensure IM delivery. Because of a finite number of commercially available needles, some participants, particularly in the low-STMD group, received IM syringe injections with needles that had a higher gauge than that used for EpiPen (22 gauge). The use of higher-gauge needles may have increased the resistance to injection, which in turn may have affected the force of injection and subsequent epinephrine PK measurements for the IM syringe group. However, the needle gauges used in the high-STMD group were the same gauge as that used for EpiPen (22 gauge) for 10 of 11 participants, so differences in needle gauge were unlikely to affect the findings in the key population of concern for this study, particularly given the consistent findings across STMD groups. Finally, this study did not use ultrasound imaging or radiolabeled injectate to assess localization of the epinephrine boluses in the muscle after epinephrine administration, so the ability of different injection methods to deliver IM epinephrine was assessed indirectly by systemic PK measurements. Despite these limitations, the broad inclusion criteria and randomized, crossover design of this study enabled investigation of multiple epinephrine administration methods across different demographics that included individuals with a compressed STMD greater than the length of the EpiPen needle.

## Conclusions

Epinephrine delivery with EpiPen may result in higher peak exposures and greater early exposure to systemic epinephrine compared with an IM syringe. Additionally, epinephrine administration via EpiPen may provide consistent systemic epinephrine delivery regardless of participants’ STMD. These findings may be confirmed with additional randomized prospective studies.

## Supplementary information

**Additional file 1:** Individual Needle Length and Gauge by Group and Sex.

**Additional file 2:** Epinephrine Pharmacokinetics After Epinephrine Injection via Different Sites and Injection Techniques.

## Data Availability

The data generated and analyzed in this study are not publicly available because of the commercially sensitive nature of the research.

## Abbreviations

AE: Adverse event
AL: Anterolateral
AUC: Area under the epinephrine plasma concentration–time curve
AUC_0-t_: AUC to the last measurable concentration
BMI: Body mass index
CI: Confidence interval
C_peak_: Peak epinephrine plasma concentration
EpiPen: EpiPen auto-injector
IM: Intramuscular
PD: Pharmacodynamics
PK: Pharmacokinetics
SC: Subcutaneous
STBD: Skin-to-bone distance
STMD: Skin-to-muscle distance
t_peak_: Time to C_peak_

## References

[1] Campbell RL, Li JTC, Nicklas RA, Sadosty AT (2014). Members of the joint task force, practice parameter workgroup. Emergency department diagnosis and treatment of anaphylaxis: a practice parameter. Ann Allergy Asthma Immunol..

[2] Simons FER, Ardusso LR, Biló MB, El-Gamal YM, Ledford DK, Ring J (2011). World allergy organization guidelines for the assessment and management of anaphylaxis. World Allergy Organ J..

[3] Muraro A, Roberts G, Worm M, Biló MB, Brockow K, Fernández Rivas M, for the EAACI Food Allergy and Anaphylaxis Guidelines Group (2014). Anaphylaxis: guidelines from the European academy of allergy and clinical immunology. Allergy..

[4] Hill RL, Wilmot JG, Belluscio BA, Cleary K, Lindisch D, Tucker R (2016). Comparison of drug delivery with autoinjector versus manual prefilled syringe and between three different autoinjector devices administered in pig thigh. Med Devices..

[5] Posner LS, Camargo CA (2017). Update on the usage and safety of epinephrine auto-injectors, 2017. Drug Healthc Patient Saf..

[6] Tsai G, Kim L, Nevis IFP, Dominic A, Potts R, Chiu J (2014). Auto-injector needle length may be inadequate to deliver epinephrine intramuscularly in women with confirmed food allergy. Allergy Asthma Clin Immunol..

[7] Song TT (2018). Epinephrine needle length in autoinjectors and why it matters. J Allergy Clin Immunol Pract..

[8] Galgatte UC, Jamdade VR, Aute PP, Chaudhari PD (2014). Study on requirements of bioequivalence for registration of pharmaceutical products in USA, Europe and Canada. Saudi Pharm J..

[9] Mean body mass index trends among adults, age-standardized (kg/m^2^): estimates by WHO region. World Health Organization. 2017. http://apps.who.int/gho/data/view.main.BMIMEANAREGv?lang=en. Accessed 5 Mar 2020.

[10] Duvauchelle T, Robert P, Donazzolo Y, Loyau S, Orlandini B, Lehert P (2018). Bioavailability and cardiovascular effects of adrenaline administered by Anapen autoinjector in healthy volunteers. J Allergy Clin Immunol Pract..

[11] Fergeson JE, Patel SS, Lockey RF (2017). Acute asthma, prognosis, and treatment. J Allergy Clin Immunol..

[12] Diacono D, Pumphrey RS, Sharma V, Arkwright PD (2015). The deep fascia of the thigh forms an impenetrable barrier to fluid injected subcutaneously by autoinjectors. J Allergy Clin Immunol Pract..

[13] Simons FE, Gu X, Simons KJ (2001). Epinephrine absorption in adults: intramuscular versus subcutaneous injection. J Allergy Clin Immunol..

[14] Song TT, Worm M, Lieberman P (2014). Anaphylaxis treatment: current barriers to adrenaline auto-injector use. Allergy.

[15] Dreborg S, Kim L, Tsai G, Kim H (2018). Epinephrine auto-injector needle lengths: can both subcutaneous and periosteal/intraosseous injection be avoided?. Ann Allergy Asthma Immunol.

[16] Dreborg S, Tsai G, Kim H (2019). Implications of variation of epinephrine auto-injector needle length. Ann Allergy Asthma Immunol.

[17] Dreborg S, Wen X, Kim L, Tsai G, Nevis I, Potts R (2016). Do epinephrine auto-injectors have an unsuitable needle length in children and adolescents at risk for anaphylaxis from food allergy?. Allergy Asthma Clin Immunol..

[18] Song TT, Nelson MR, Chang JH, Engler RJ, Chowdhury BA (2005). Adequacy of the epinephrine autoinjector needle length in delivering epinephrine to the intramuscular tissues. Ann Allergy Asthma Immunol.

[19] United Kingdom (UK) Resuscitation Council. Emergency treatment of anaphylactic reactions: guidelines for healthcare providers. 2008. https://www.resus.org.uk/EasySiteWeb/HandleRequest/_resources/assets/attachment/full/0/824.pdf. Accessed 5 Mar 2020. 10.1016/j.resuscitation.2008.02.001 18358585

[20] Song TT, Lieberman P (2016). Epinephrine auto-injector needle length: what is the ideal length?. Curr Opin Allergy Clin Immunol.

