# Supplementary material for: Epinephrine delivery via EpiPen® Auto-Injector or manual syringe across participants with a wide range of skin-to-muscle distances
Source: Clin Transl Allergy. 2020 Jun 10;10:21. doi: 10.1186/s13601-020-00326-x (PMC7285563; doi:10.1186/s13601-020-00326-x)
Supplement: Supplementary file 1 — Additional file 1: Individual Needle Length and Gauge by Group and Sex. [file 13601_2020_326_MOESM1_ESM.docx]

| **Additional file 1.** Individual Needle Length and Gauge by Group and Sex^a^ | | |
| --- | --- | --- |
| **Sex** | **Needle length, mm** | **Gauge** |
| **Low STMD (<15 mm; n=12)** | | |
| F | 12 | 26 |
| F | 12 | 26 |
| F | 20 | 27 |
| F | 25 | 23 |
| F | 25 | 23 |
| F | 30 | 23 |
| M | 12 | 26 |
| M | 16 | 25 |
| M | 16 | 25 |
| M | 20 | 27 |
| M | 20 | 27 |
| M | 25 | 23 |
| **Moderate STMD (15-20 mm; n=12)** | | |
| F | 25 | 23 |
| F | 25 | 23 |
| F | 25 | 23 |
| F | 25 | 23 |
| F | 30 | 23 |
| F | 30 | 23 |
| M | 25 | 23 |
| M | 25 | 23 |
| M | 25 | 23 |
| M | 30 | 23 |
| M | 30 | 23 |
| M | 40 | 22 |
| **High STMD (>20 mm; n=11)** | | |
| F | 30 | 23 |
| F | 40 | 22 |
| F | 40 | 22 |
| F | 40 | 22 |
| F | 40 | 22 |
| F | 40 | 22 |
| M | 40 | 22 |
| M | 40 | 22 |
| M | 40 | 22 |
| M | 40 | 22 |
| M | 40 | 22 |
| F, female; M, male; STMD, skin-to-muscle distance. ^a^The following syringes were available for the study: 10 mm × 26 gauge, 12 mm × 26 gauge, 16 mm × 25 gauge, 20 mm × 27 gauge, 25 mm × 23 gauge, 30 mm × 23 gauge, and 40 mm × 22 gauge. No needle length beyond 40 mm was required for this study. | | |
