# Supplementary material for: Epinephrine delivery via EpiPen® Auto-Injector or manual syringe across participants with a wide range of skin-to-muscle distances
Source: Clin Transl Allergy. 2020 Jun 10;10:21. doi: 10.1186/s13601-020-00326-x (PMC7285563; doi:10.1186/s13601-020-00326-x)
Supplement: Supplementary file 2 — Additional file 2: Epinephrine Pharmacokinetics After Epinephrine Injection via Different Sites and Injection Techniques. [file 13601_2020_326_MOESM2_ESM.docx]

| **Additional file 2.** Epinephrine Pharmacokinetics After Epinephrine Injection via Different Sites and Injection Techniques | | | |
| --- | --- | --- | --- |
| **Parameter** | **EpiPen,  mid-AL thigh  (N=35)** | **IM syringe,  mid-AL thigh  (N=35)** | **EpiPen,  distal-AL thigh  (n=23)** |
| C_peak_ | | | |
| Mean (SD), ng/mL | 0.52 (0.26) | 0.35 (0.15) | 0.41 (0.24) |
| CV, % | 51 | 43 | 58 |
| Geomean, ng/mL | 0.45 | 0.32 | 0.35 |
| t_peak_ | | | |
| Mean (SD), min | 23 (24) | 43 (16) | 27 (18) |
| Median (range), min | 20 (2-120) | 50 (3-60) | 25 (3-60) |
| CV, % | 102 | 37 | 67 |
| Geomean, min | 14 | 37 | 21 |
| AUC_0-t_ | | | |
| Mean (SD), ng·min/mL | 30.0 (12.4) | 26.1 (10.3) | 28.1 (12.3) |
| CV, % | 41 | 39 | 44 |
| Geomean, ng·min/mL | 27.1 | 23.9 | 25.2 |
| AL, anterolateral; AUC_0-t_, area under the epinephrine plasma concentration-time curve to the last measurable concentration; C_peak_, peak epinephrine plasma concentration; CV, coefficient of variation; EpiPen, EpiPen Auto-Injector; geomean, geometric mean; IM, intramuscular; SD, standard deviation; t_peak_, time to C_peak_. | | | |
